# Role of Early Maladaptive Schemas on Addiction Potential in Youth

**DOI:** 10.5812/ijhrba.10148

**Published:** 2013-09-20

**Authors:** Fereshteh Bakhshi Bojed, Zahra Nikmanesh

**Affiliations:** 1 Department of Psychology, Faculty of Education and Psychology, University of Sistan and Baluchestan, Zahedan, IR Iran

**Keywords:** Behavior, Addictive, Adolescent

## Abstract

**Background:**

Drug abuse in juveniles is one of the most serious problems which lead to different physical, social and educational damages and outcomes.

**Objectives:**

The aim of the present study is to predict the addiction Potential in youths by their early maladaptive schemas.

**Materials and Methods:**

The research sample included students of the University of Sistan and Baluchistan, Zahedan, Iran, with average age of 19-24 years. Participants were 260 undergraduate students (159 girls and 101 boys) that were selected by probability proportional cluster random sampling. The instruments were the Addiction Potential Scale (APS) and Early Maladaptive Schemas SQ-SF (short form questionnaires).

**Results:**

The result showed that there are positive and significant relationships among early maladaptive schemas include Disconnection/ Rejection, Impaired autonomy / Performance, Impaired Limits, Other-Directedness and Over vigilance / Inhibition, and addiction Potential. Also, results of regression showed the highest Addiction Potential predicted by the following schemas: Disconnection / Rejection, impaired autonomy and performance other-Directedness.

**Conclusions:**

Therefore, Addiction Potential is one of the areas which can be used in planning and preventive activities, through identifying the groups with Early Maladaptive Schemas.

## 1. Background

Addiction is a physical, mental, social and psychic disease where various pre-addiction factors play a basic role in its formation (1). "Addiction Potential" is defined as the beliefs and attitudes of people about drugs, and the negative and positive outcomes of using them (2). Tendency is an internal feeling with high probability of shaping some behaviors or simply learning them (3). It was shown that drug users suffer from some early maladaptive schemas which can be the Potential for drugs abuse (4). Schemas are formed from early life and affect people throughout their lifespan (5). Early maladaptive schemas are the kind of beliefs that people have about themselves, others, and the environment which are normally derived from dissatisfaction about basic needs, especially emotional needs in childhood (6). When the early maladaptive schemas become active, the levels of released excitement and direct or indirect excitement lead to various types of cognitive disorders such as depression, anxiety, occupational disability, lack of academic progress, drug abuse, and interpersonal conflicts (7). Young, Klosko and Weishour identified eighteen early maladaptive schemas and they introduced them in five areas as follows: Disconnection/Rejection (abandonment/instability, mistrust/abuse emotional deprivation, defectiveness/shame, social isolation/alienation); Impaired autonomy/Performance (dependence/incompetence, vulnerability to harm or illness, enmeshment/undeveloped self, failure); Impaired Limits: (entitlement/grandiosity, insufficient self-control/self-discipline); Other directedness: (subjugation, self-sacrifice, approval-seeking/recognition-seeking); and Over vigilance/Inhibition (negativity/pessimism, emotional inhibition, unrelenting standards/hypocriticalness and punitiveness) (8). Young believes that maladaptive schemas result in experiencing the negative events in life and these negative events cause irregular psychic pressures in people (9). Then these people, who use maladaptive schemas inordinately, are affected more by negative events (10). Young et al. stated that: "people with positive schemas are at lower risk of illness because they experience more positive excitements, and when they encounter problems, they show more strength at coping" (8). Coping responses are usually used for controlling early maladaptive schemas and expressing a kind of avoiding behavior which can result in drug abuse as a defense mechanism (11). Authors found no studies which directly investigate the relationship between early maladaptive schemas and addiction Potential. But in the literature, the relationship of schemas with non-addicts and addicts was studied. Rake, Boer and De boa argued that "people who use adaptive schemas have more capabilities to cope with mental pressures and when they encounter stressing events, they are less likely to suffer from mental problems and drug abuse (12). Findings showed that maladaptive schemas in drug users are higher than the other people (13-15). In another research conducted on alcohol addiction, it was shown that most of the alcoholics have more early maladaptive schemas in comparison with normal people (16).

Also, the results showed that drug users apply Disconnection/Rejection schemas (17). A study showed that people with dependence/incompetence and defectiveness/shame schemas have a tendency to use drugs (18). In another research, it was indicated that personality troubles and addiction mostly appear as emotional deprivation, dependence/incompetence, entitlement/grandiosity, enmeshment/undeveloped self and failure schemas (19). Another study, on a group of 196 psychiatric patients, showed that maladaptive schemas are the most efficient predictors of mental disorders as anxiety, depression, paranoia and drug abuse (20).

Therefore, the disability in coping with stress and this belief that using drugs will have desirable outcomes are the basis for addiction potential (21). Accordingly, the current study aims to answer the following research questions: 1) Is there a relationship between Addiction Potential and Early Maladaptive Schemas areas among the youth? 2) Can early maladaptive schemas predict the addiction potential?

## 2. Objectives

The aim of the present study is to predict the Addiction Potential according to the Early Maladaptive Schemas in youths.

## 3. Materials and Methods

The method of this research is descriptive and correlation type. The Statistical population consisted of students in year 2011-2012 in the University of Sistan and Baluchestan. The sample was comprised of 260 subjects (159 female and 101 male) that were selected using the proportional cluster method. Age range of the subjects was from 19 to 25 years. The data was analyzed by Pearson Correlation and Stepwise regression.

### 3.1. Research instruments

1. Addiction Potential Scale (APS): Inventory of Addiction Potential Scale (APS) was used to evaluate the addiction potential. The APS subscale of Weed et al. (22) consisted of three addiction potential subscales, addiction acknowledgment scales (AAS), and Mc Andrew scale of potential for wine drinking was used. The APS consists of 39 questions. The response to each question was either “yes” or “no”. Weed et al. (22) have obtained the coefficients of reliability of the ARS in a normal sample for men and women 0.69 and 0.77, respectively. In the present research, the reliability coefficient of the inventory with a sample size of 67 subjects was 0.80.

2. Young Schema Questionnaire–Short Form (YSQ-SF: Young, 1998): The YSQ-SF consists of 75 items assessing 16 early maladaptive schemas. Each scale consists of five items rated on a six-point scale. A description of the 16 scales is provided below: Disconnection/Rejection (abandonment/instability, mistrust/abuse, emotional deprivation, defectiveness/shame, social isolation/alienation); Impaired autonomy/Performance (dependence/incompetence, vulnerability to harm or illness, enmeshment/undeveloped self, failure); Impaired Limits (entitlement/grandiosity, insufficient self-control/self-discipline); Other Directedness (subjugation, self-sacrifice, approval-seeking/recognition-seeking); and Over vigilance/Inhibition (negativity/pessimism, emotional inhibition, unrelenting standards/hypocriticalness and punitiveness) (8). The factor structure has been supported and further developed by heretical factor analysis. Considerable internal consistency has been found for all 16 of the schema scales. These coefficients ranged from 0.73 to 0.93 (23, 24).

## 4. Results

Descriptive results of this research are presented below. Table 1 shows that the mean and standard deviation of addiction potential among female students are less than those among male students. 

**Table 1.** Mean and Standard Deviation of Drug Usage

Gender	Frequency	Mean ± SD
**Girl**	101	18.03 ± 4.10
**Boy**	159	19.67 ± 4.44
**Total**	260	19.03 ± 44.37

**Table 2.** Mean and standard deviation early Maladaptive Schemas (N = 260)

Early Maladaptive Schemas	Mean ± SD
**Disconnection/rejection**	64.72 ± 22.83
**Impaired autonomy and performance**	45.28 ± 17.46
**Impaired limits**	32.66 ± 9.37
**Other-directedness**	28.81 ± 8.84
**Over vigilance/inhibition**	37.21 ± 10.85

Results are presented according to the research questions posed below:

1) Is there a relationship between addiction potential and Early Maladaptive Schemas areas among the youth? (Table 2)

The result of Pearson correlation shows that there are positive relationship among the addiction potential and all of the five areas of early maladaptive schemas (Disconnection/Rejection, impaired autonomy and performance, Impaired Limits, Other- Directedness and Over vigilance/Inhibition). These relations were significant at 0.01.

2) Which one of the early maladaptive schema areas is a stronger predictor of addiction potential among the youth? (Table 3)

The results in Table 3 showed, in the first step of regression analysis, the schema of Disconnection/Rejection alone determined 0.16 of the variances of the addiction potential. In the second step, the schema of impaired autonomy and performance variables enter into the prediction model and along with the previous variable predicted 172 of the addiction potential variation. In the third step, the schema of other directedness with the previous two variables explained 0.182 of the variation. In addition, the Beta coefficients for the schemas of Disconnection/Rejection, impaired autonomy and performance and other-directedness, respectively, are 0.366, 0.205, 0.144. 

**Table 3.** Summary of the Regression Model for Prediction Drug Usage

Step	Variables	R^*^	R^2^	β	F	Sig.
**1**	**Disconnection/Rejection**	0.16	0.15	0.40	49.19	0.000
**2**	**Disconnection/Rejection**	0.17	0.17	0.30	5.66	0.000
	Impaired autonomy/Performance			0.16		
**3**	**Disconnection/Rejection**			0.36		
	Impaired autonomy/Performance	0.19	0.18	0.20	3.94	0.000
	Other directedness			0.14		

^*^Abbreviations: R, Relation; B, Beta; Sig, Signification

## 4. Discussion

Early maladaptive schemas play an important role in the mentality, feelings and performance of people or the way they communicate with each other (8). Early maladaptive schemas and maladaptive styles, which people learn how to encounter them, are almost the basis for chronic disorder factors such as drug abuse disorders, depression, anxiety and psychosomatic disorders (5). This finding goes along with the theory of Young schema that assumes maladaptive schemas directly or indirectly cause the problems and psychological disorders and behaviors like alcoholism and drug addiction. According to Young, maladaptive behaviors are created in response to schemas and then these behaviors are excited by the same schemas; and when the maladaptive schemas are excited, people experience high levels of (negative) feelings such as severe resentment, anxiety, distress or feeling guilty. This severity of excitement is usually unpleasant, therefore, people almost use maladaptive behaviors such as abusing drugs in order to avoid exciting the schemas and of the feeling of excitement associated with these schemas (8). Therefore, drug abuse in adolescent and youth is one of the most severe problems that lead to different physical, social and educational problems and outcomes. the outcomes of drug abuse at the social level include: reduced motivation, mental and cognitive disorders, temperamental disorders, physical damages and delinquency, educational failures, interpersonal relationship disorders (7).

According to the results of the current study, there is a significantly positive relationship among the five areas of early maladaptive schemas: Disconnection/Rejection, impaired autonomy and performance, Impaired Limits, Other-Directedness and Over vigilance/Inhibition, and the addiction potential. In this context, the schemas Disconnection/Rejection, impaired autonomy and performance, and Other-Directedness had the highest prediction of the addiction potential variances. Accordingly, we can infer that people with addiction potential has early maladaptive schemas. Some of the studies have pointed to the correlation between early maladaptive schemas and drug dependence (14, 25), and this supports the preceding results. For example, Ball et al. had assumed that early maladaptive schemas appeared in the area of Disconnection/Rejection especially in the drug abusing group (12). Another study found that having early maladaptive schema is predictive of interpersonal maladjustment (5). Also, the present results coincide with many of the previous studies (4, 13-17). They indicated that addiction is correlated with early maladaptive schemas. Furthermore, these studies showed that the abundance of the schemas of Disconnection/Rejection area (abandonment/instability, mistrust/abuse, emotional deprivation, defectiveness/shame, social isolation/alienation) is greater than other areas, which is true for the findings of the current study.

Moreover, the regression results indicated that maladaptive schemas in Disconnection/Rejection area are the strongest predictors for addiction potential. Similarly, researchers showed that early maladaptive schemas, especially in areas of Disconnection/Rejection schemas, impaired autonomy and performance, and other directions play an important role in the prediction of addiction (18-20). Cognitive schemas in Disconnection/Rejection area show that personal needs are not satisfied with safety, stability, affection, sympathy, sharing feelings, acceptance and respect in predictable styles. These kinds of schemas are usually grown in the insensitive, cold, withholder, secluded, acrid, and unpredictable or misconduct families. Cognitive schemas in the impaired autonomy and performance indicate an inconsistency between expectations from oneself and environment with his/her abilities in actual performance. This area of schemas is formed with in the families that reduce the self-confidence of the child, protect the child excessively or could not encourage the child to do outdoor activities. Other direction schema indicates that these people ignore his/her needs and extremely focus on the desires, feelings and responses of others (8).

It can be concluded that the strategies to prevent the addiction potential need to achieve adaptive schemas. From the applied point of view, instruction for positive and adaptive schemas creates better feelings and capability in people, and enables them to better cope with problems. In addition, addiction potential is one of the areas which can be used in preventive planning. Moreover, it is useful to identify the groups with high tendency towards addiction and creating suitable preventive and therapeutic strategies. Ball and Young cited that therapeutic schema plays an important role in successful addiction treatment (26).

According to the age limit of subjects in this study, generalization of the present results to other age groups should be made cautiously.
